# Addressing astringency of grape seed extract by covalent conjugation with lupin protein

**DOI:** 10.1016/j.crfs.2024.100795

**Published:** 2024-06-17

**Authors:** Cristhian Rafael Lopes Francisco, Siavash Soltanahmadi, Tatiana Porto Santos, Rosiane Lopes Cunha, Anwesha Sarkar

**Affiliations:** a Food Colloids and Bioprocessing Group, School of Food Science and Nutrition, Faculty of Environment, University of Leeds, Leeds, LS2 9JT, UK; b Laboratory of Process Engineering, Department of Food Engineering and Technology, School of Food Engineering, University of Campinas (UNICAMP), Rua Monteiro Lobato 80, 13083-862, São Paulo, Campinas, Brazil; c Laboratory of Food Process Engineering, Wageningen University and Research, Bornse Weilanden 9, 6708 WG, Wageningen, the Netherlands

**Keywords:** Conjugation, Proanthocyanidin, QCM-D, Tribology, Plant protein

## Abstract

Astringency of phenolic-rich foods is a key tactile perception responsible for acceptability/rejection of plant extracts as ingredients in formulations. Covalent conjugation of phenolic extracts with plant proteins might be a promising strategy to control astringency, but suffers from a lack of mechanistic understanding from the lubrication point of view. To shed light on this, this *ex vivo* study evaluated the effect of conjugation of a phenolic grape seed extract (GSE) with legume protein (lupin, LP) on tribological and surface adsorption performance of GSE in the absence and presence of human saliva (*ex vivo*). Tribological results confirmed GSE had an inferior lubrication capacity as compared to LP. The lubrication performance of LP-GSE dispersions was comparable to their corresponding LP dispersion (*p > 0.05*) when covalently conjugated with LP (LP-GSE) with increasing LP:GSE ratio up to 1:0.04 w/w and at a specific degree of conjugation (DC: 2%). Tribological and surface adsorption measurements confirmed the tendency of GSE to interact with human saliva (*ex vivo*, n = 17 subjects), impairing the lubricity of salivary films. The covalent bonding of LP to GSE hindered GSE's interaction with human saliva, implying the potential influence of covalent conjugation on attenuating astringency. LP appeared to compete with human saliva for surface adsorption and governed the lubrication behaviour in LP-GSE dispersions. Findings from this study provide valuable knowledge to guide the rational design of sustainable, functional foods using conjugation of phenolics with plant proteins to incorporate larger proportions of health-promoting phenolics while controlling astringency, which needs validation by sensory trials.

## Highlights

• Grape seed extract (GSE) reduced the lubrication performance of saliva.
• Friction coefficient of GSE can be decreased by conjugation with lupin protein (LP).
• Degree of conjugation modulated the frictional behaviour of LP-GSE conjugates.
• Conjugation of LP to GSE reduced salivary protein precipitation *via* GSE.
• Adsorption data confirmed limited interaction of conjugate with salivary proteins.

## Graphical abstract

**Figure:**
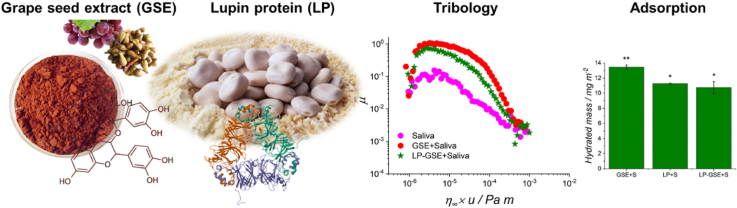

## 1 Introduction

Plant-sourced phenolic (PC) compounds such as phenolic acids, flavonoids, and tannins (Vuolo et al., 2019) have been in the research spotlight for the past few decades due to their potential health-promoting properties, such as antimicrobial, antioxidant, anti-inflammatory, anticancer (Camboim Rockett et al., 2020; Chlif et al., 2022; Duan et al., 2020; García-Lafuente et al., 2014; Küpeli et al., 2007; Mumtaz et al., 2021; Pereira et al., 2007; Su et al., 2009; Xu et al., 2018), evaluated from *in vitro* and *in vivo* (animal and cell models) studies. However, PC-rich foods often suffer from an unpleasant mouthfeel perception of tactile origin named astringency, a feeling of dryness associated with disturbed salivary lubrication. Astringency caused by PCs is a complex sensorial/neural phenomenon that can be explained in parts by the loss of oral lubricity caused mainly by the non-covalent interaction of hydroxyl groups in PCs with salivary proteins forming precipitated complexes (Huang and Xu, 2021; Ma et al., 2014). Often, to control such astringency perception in PC-rich beverages such as wine, fining agents (*e.g.,* proteins) are used (Cosme et al., 2007; Ricardo-da-Silva et al., 1991). Plant proteins have been used as fining agents with the hypothesis that covalent or non-covalent interactions (Chantal et al., 2003; Marangon et al., 2019) of plant proteins with hydroxyl groups in PCs desirably occupying the binding sites limit the interaction of PCs with salivary proteins (Quan et al., 2019). Nevertheless, a systematic probing into the physics of how a plant protein may prevent PC from interacting with salivary proteins remains overlooked in literature.

Grape seed extract (GSE) is a health promoting PC compound, a by-product in grape juice and wine processing originating from grape seeds (*Vitis vinifera*) following extraction, drying, and purification post-processing to enhance its content of PC (Lau and King, 2003). GSEs stand out from other commercially available PC extracts because of their higher concentration of oligomeric proanthocyanidins at proportions more than 90% (Hagerman et al., 1998; Nakamura et al., 2003; Ricci et al., 2017). Oligomeric proanthocyanidins are composed of a class of condensed tannins well known for their potential biofunctional properties (tested in *in vitro* and *in vivo* studies) such as wound healing, anti-inflammatory, antioxidant, cardiovascular, antihypertensive, antiulcer, antimicrobial, and anticancer activities (Gupta et al., 2020), though well known for their astringent perception (Ma et al., 2014; Monteleone et al., 2004). Lupin protein (LP) on the other hand has gained worldwide interest as a plant protein due to its nutritional value, low environmental footprints, and low-cost cultivation (Shrestha et al., 2021). Francisco et al. (2023) have shown promising covalent interaction between the proanthocyanidins from GSE and LP, indicating the potential of LP to act as a proanthocyanidin fining agent (Chantal et al., 2003). A quantitative understanding of how LP interact with GSE to reduce further interactions with human saliva may be deciphered using oral tribology and surface adsorption studies which is missing in the literature.

Oral tribology can successfully be used as a quantitative, physical proxy to study the astringency of PCs (Laguna and Sarkar, 2017) and to correlate the sensory attributes (*i.e.*, slipperiness, smoothness, pastiness, melting, dryness) with friction coefficient measurements across the lubrication regimes (*i.e.*, boundary, mixed and hydrodynamic) (Pradal and Stokes, 2016; Sarkar and Krop, 2019). Studies have reported a decrease in salivary lubrication (*i.e.*, an increase in friction coefficient) in the presence of PCs, corroborating sensorial trials (Rudge et al., 2021; Wang et al., 2020, 2021). Previous tribological studies evaluated the astringency of wines (Wang et al., 2020, 2021) and have measured the lubrication and adsorption performance of LP (Kew et al., 2021; Liamas et al., 2023; Kew et al., 2021; Liamas et al., 2023). However, a systematic quantification of tribological and adsorption properties of LP-GSE conjugates and their synergy in interaction with human saliva have not been performed in the literature to date. In this context, such a study may offer valuable *in vitro* information on how the conjugation of PCs with plant protein can affect the sensory perception of GSE while retaining GSE for its health benefits.

Therefore, this study addresses this knowledge gap by unveiling the physics of conjugating LP to GSE on frictional performance of PCs’ interaction with saliva. To first understand the role of the LP and GSE on the lubrication performance of conjugates, two aspects of their formulation were varied: (i) the LP:GSE ratio and (ii) the degree of conjugation and were compared against control samples (LP and GSE dispersions). The surface adsorption measurements were performed *via* the quartz crystal-microbalance with dissipation (QCM-D) technique to provide insights into the nature of interactions between biopolymers (GSE, LP and LP-GSE) and human saliva/surfaces.

## 2 Material and methods

### 2.1 Material

Lupin protein isolate (LP, composition: 93 wt% (protein) and 7 wt% (fiber + ash) on a dry basis, (Vogelsang-O’Dwyer et al., 2020) was kindly donated by the Fraunhofer-Institut für (Freising, Germany). Grape seed extract (GSE, 95 wt% of proanthocyanidins) was purchased from Luna Ervas (Caieiras, Brazil) with the batch number #HK20082601. Pierce™ Bradford Protein Assay Kit was purchased from Thermo Fisher Scientific (Waltham, USA). 4-(2-hydroxyethyl)-1-piperazineethanesulfonic acid (HEPES) was purchased from ITW Reagents (UK). The other chemicals and solvents were of analytical grade. Milli-Q water (18.2 MΩ cm at 25 °C, Millipore Corp., Bedford, MA, USA) was used in all assays.

### 2.2 Methods

#### 2.2.1 Preparation of biopolymer dispersions and conjugates dispersions

Conjugation of LP to GSE was carried out using a procedure described by Francisco et al. (2023) with some modifications. In order to evaluate the effect of LP concentration on the lubrication behaviour of LP-GSE conjugates, LP at concentrations of 1, 3, 5, and 11 wt% were mixed at a fixed concentration of GSE (0.12 wt%), resulting in LP:GSE ratios of 1:0.12, 1:0.04, 1:0.02, 1:0.01 w/w, respectively. These correspond to molar ratios of 1:42, 1:14, 1:8, and 1:4 mol/mol with consideration of the molecular weight of globulins (Duranti et al., 2008) and proanthocyanidins A as the main composition of LP and GSE, respectively (National Center for Biotechnology Information, 2023). To obtain LP dispersions at 1, 3, 5, and 11 wt%, LP was firstly dissolved at room temperature in Milli-Q water at a pre-defined concentration (natural pH of about 6.5 for 2 h). Then, the LP dispersions were centrifuged at 8000 *g* for 10 min to remove any insoluble fractions. The final concentration of LP (*i.e.,* 1, 3, 5, and 11 wt%) was verified using a Bradford Protein Assay Kit. To promote the conjugation, the pH of the protein dispersion was adjusted to pH 9.0 using 1.0 M NaOH, followed by the addition of GSE to make up a 0.12 wt% GSE in LP dispersions and mixing for a duration of 4 h under atmospheric conditions. Following the conjugation, the pH of the dispersions was adjusted to pH 7.0 (1.0 M HCl) for further analysis. In order to evaluate the effects of the degree of conjugation on the lubrication behaviour of LP-GSE, the conjugation was conducted at 4, 24, 48, and 168 h and a LP:GSE ratio of 1:0.04 w/w. Control samples containing LP (1, 3, 5, and 11 wt%) were obtained by adjusting the pH to pH 9.0 and mixing for a specific time to match the time of conjugate production, and finally adjusting the pH to 7.0 before the measurements. This procedure was adapted to minimize the effects of pH on the protein structure when comparing LP to LP-GSE samples. Control samples of GSE (0.12, 0.5, and 1 wt%) dispersed in MilliQ water were also prepared. Sodium azide (0.02 wt%) was used as an antimicrobial agent for all dispersions. Samples were named to represent the concentration of LP and GSE and the degree of conjugation in the formulation determined using *O*-phthalaldehyde (OPA assay, discussed later), as listed in Table 1.

**Table 1:** Sample nomenclature specifying the composition of the dispersions and degree of conjugation.

Family of samples	Sample name	Lupin protein (wt%)	Grape seed extract (wt%)	Degree of conjugation (%)
Biopolymer	GSE[0.12]	–	0.12	–
	GSE[0.5]	–	0.5	–
	GSE[1]	–	1	–
	LP[1]	1	–	–
	LP[3]	3	–	–
	LP[5]	5	–	–
	LP[11]	11	–	–
Conjugate^a^	LP[1]-GSE	1	0.12	–
	LP[3]-GSE	3	0.12	–
	LP[5]-GSE	5	0.12	–
	LP[11]-GSE	11	0.12	–
Conjugate^b^	LP-GSE_DC[2]_	3	0.12	2
	LP-GSE_DC[6]_	3	0.12	6
	LP-GSE_DC[7]_	3	0.12	7
	LP-GSE_DC[12]_	3	0.12	12

a Family of samples in which lupin protein concentration was varied.

b Family of samples in which the degree of conjugation was varied.

### 2.3 Characterization of LP-GSE conjugates

#### 2.3.1 Degree of conjugation (DC)

The free amino groups of LP and LP-GSE conjugates were measured *via* the OPA assay (Church et al., 1983) using a UV/Vis spectrophotometer (CE9500, Cecil Instruments Ltd, UK) and *O*-phthalaldehyde reagent. Prior to the analysis, the dispersions were centrifuged (5000 *g* for 5 min) (Heraeus Fresco 21, Thermo Scientific, USA) to remove the insoluble fraction of GSE. The concentration of free amino groups was determined, and the DC was calculated as described in Equation (1).

Eq. 1 $$DC\left(\%\right)=\frac{C_{Lupin}-C_{conjugate}}{C_{lupin}}x100$$

where, C_Lupin_ and C_Conjugate_ correspond to the concentration of free amino groups present in pure lupin protein and conjugate samples, respectively (Soltanahmadi et al., 2023).

#### 2.3.2 Collection of saliva

Unstimulated human saliva samples were collected no more than 72 h prior to each experiment. Participants were informed about the study and asked to sign a consent form (Faculty Research Ethics Committee of the University of Leeds (MEEC-19-028)). Male and female participants (n = 17) from 22 to 49 years old were asked to abstain from eating and drinking (except water) for 2 h before the collection of saliva to reduce the influence of the intake of foods and beverages. During saliva collection (between 10 a.m. and 12 p.m.), participants were asked to passively accumulate saliva in the mouth and then spit it into a clean tube kept on ice during the collection time (15 min). Immediately after collection, saliva samples from all the participants were pooled and centrifuged (10 min at 4000 g). The supernatant was collected and stored at 4 °C before use. It is expected that the lubricating properties of human saliva are less sensitive to saliva treatment (*e.g.* centrifugation) than its bulk viscoelastic properties (Bongaerts et al., 2007).

#### 2.3.3 Preparation of saliva mixtures

For studying the interaction of GSE, LP, and LP-GSE with saliva, GSE[0.12], LP[3], and LP[3]-GSE dispersions in HEPES (20 mM, pH 7) were mixed with saliva in a sample: saliva ratio of 4:1 w/w for 5 min immediately before measurements to simulate the dilution during oral processing (Devezeaux de Lavergne et al., 2015). A solution with water: saliva ratio of 4:1 w/w was used as the control sample.

#### 2.3.4 Bulk rheological measurements

The apparent viscosity (*η*) of biopolymers, conjugates, and saliva mixture samples was measured by a modular compact controlled-stress rheometer (MCR- 302, Anton Paar, Austria using a double gap geometry (DG27) at 37 °C to mimic physiological temperature. *η* values were measured at shear rates ($\dot{\gamma }$) ranging from 10^−1^ to 10^2^ s^−1^, ramped logarithmically to record five data points within each decade. At each $\dot{\gamma }$, a minimum 30 s window was set to achieve stress stability.

### 2.4 Lubrication performance

A mini traction machine (MTM2, PCS instruments, UK) in a ball-on-disk configuration was used to measure friction coefficients (*μ*) as a function of the entrainment speed (*u*). Tribological measurements were conducted using polydimethylsiloxane (PDMS) specimens (ball 19 mm and disc 46 mm in diameter) at 37 °C and a slide-to-roll ratio (SRR) of 0.5. A normal load of 2 N was used, equal to a maximum Herzian contact pressure of ∼200 kPa (Sarkar et al., 2019b) with speed ranging from 10^−3^ to 3 m/s. To better reflect on the effect of interactions between biopolymers and PDMS surfaces on their frictional behaviour, the *μ* curves were plotted against the reduced speed parameter, *i.e.*, *η*_*∞*_ *x u*. The viscosity value at a shear rate of 10^3^ s^−1^ was selected as *η*_*∞*_ considering the Newtonian behaviour of all dispersions (de Vicente et al., 2005; Soltanahmadi et al., 2022).

For statistical analysis, *μ* values were taken from the boundary, mixed, and hydrodynamic regimes. For the boundary regime, *μ* was taken where a plateau was observed at the region of *u* < 10^−2^ m s^−1^ (at *η*_*∞*_ *x u* ranging from about 3 to 8 × 10^−6^ P m, depending on the sample). For the mixed regime, an arbitrary *μ* value was taken at *η*_*∞*_ *x u* of 5 × 10^−5^ Pa m to represent this regime, and for the elasto-hydrodynamic regime, *μ* was determined where the lowest value was observed. The PDMS specimens were cleaned by sequential sonication steps in sodium dodecyl sulfate (2 wt% in deionized water), isopropanol alcohol, and deionized water for 10 min at each step between each measurement.

### 2.5 Quartz crystal microbalance with dissipation monitoring (QCM-D)

A QCM-D apparatus (E4 system, Q-Sense, Biolin Scientific, Sweden) was used to measure the hydrated adsorbed mass of LP, GSE, LP-GSE conjugate, saliva, and their combination, and to assess the viscoelastic properties of the adsorbed films. PDMS-coated quartz sensors were used, which were prepared by spin-coating silica sensors (QSX-303, Q-Sense, Biolin Scientific, Sweden) with 0.5 wt% PDMS solution in toluene. The spin coating was carried out at 5000 rpm for 60 s with an acceleration rate of 2500 rpm/s before leaving overnight in a vacuum oven at 80 °C (Zembyla et al., 2021). Prior to use, the PDMS-coated crystals were cleaned by immersing them in toluene for 30 s, then 30 s in isopropanol, and lastly in water for 5 min, followed by drying under nitrogen gas.

LP, GSE, and LP-GSE conjugate (LP[3]-GSE) samples were produced and equilibrated (25 °C) in 20 mM HEPES buffer in a concentration of 0.1 mg/mL for measurements without saliva and 1 mg/mL for measurements involving a pre-adsorbed salivary film. Saliva was diluted to a final protein concentration of 0.1 mg/mL. The flow rate was controlled using a peristaltic pump at 100 μL/min at 25 °C. The buffer solution was initially injected to obtain a stable baseline reading. Then, the prepared dispersions were injected until an equilibrium was reached, *i.e.*, no change in frequency (*f*) or dissipation (*D*) signals. Finally, the buffer was injected to remove loosely adsorbed layers. To calculate the hydrated mass of the adsorbed films and their viscoelasticity (*-ΔD/Δf*), the frequency data were fitted to viscoelastic Voigt's model (Voigt, 1889) using the incorporated software (Smartfit Model by Dfind, Q-Sense, Biolin Scientific, Sweden). The 3rd, 5th, 7th, and 9th overtones were considered for data analysis, and only the 5th overtone is shown in the results. A minimum of three replicates were obtained for each sample.

### 2.6 Statistical analysis

All measurements were performed at least three times in triplicate dispersions. Results were expressed as mean ± standard deviation. The data obtained were statistically assigned by one-way analysis of variance (ANOVA) and significance was confirmed through a Tukey test (significance level of 5%). The software OriginPro 2018 was used for statistical analysis and graph plotting.

## 3 Results and discussion

### 3.1 Bulk rheological measurements

**Fig. 1:**
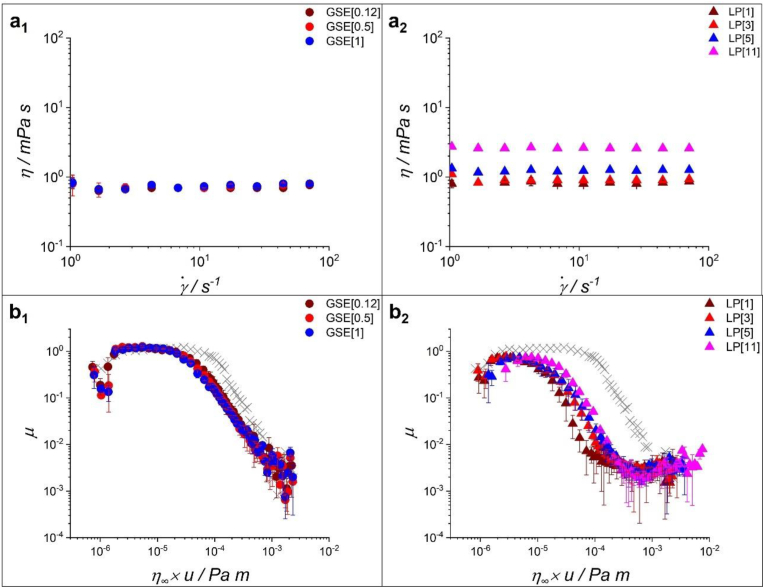
Flow curves of (a_1_) grape seed extract (GSE) dispersions and (a_2_) lupin protein (LP) dispersions and friction curves normalized to the viscosity at a shear rate of 10^3^ s^−1^ (*η*_*∞*_) for (b_1_) GSE dispersions, and (b_2_) LP dispersions. The gray symbol is the curve for buffer. Error bars represent standard deviations (n = 3 × 3).

The flow curves for the dispersions of GSE (GSE[0.12], GSE[0.5], and GSE[1]) and LP (LP[1], LP[3], LP[5], and LP[11]) are shown in Fig. 1a_1_ and a_2_. All the dispersions of GSE and LP showed a Newtonian behaviour at shear rates below 10^2^ s^−1^. The viscosity values remained almost identical and close to that of water (∼1 mPa s) (*p < 0.05*) as the concentration of GSE increased from 0.12 to 1 wt% (Fig. 1a_1_), suggesting a lack of any measurable particle-particle interactions in GSE dispersions within the experimentally tested concentrations. LP dispersions (Fig. 1a_2_) demonstrated a concentration-dependent behaviour with a slight increase in viscosity (0.8–3 mPa s) up to a concentration of 11 wt%. Conjugates at different LP:GSE ratios showed similar viscosity values to the respective LP dispersions at the same protein concentration (data not shown). Likewise, the increase of DC did not change the viscosity of LP[3]-GSE conjugates, with all samples showing similar viscosity values (data not shown).

### 3.2 Lubrication performance

#### 3.2.1 Lubrication behaviour of sole components

First, we characterized the tribological performance of the sole components *i.e.,* GSE and LP dispersions, to understand how friction changes when they are conjugated. Fig. 1b_1_ and b_2_ show the *μ - η*_*∞*_ *x u* curves for the GSE and LP dispersions, respectively. Details on *μ* values for all the dispersions at boundary, mixed and hydrodynamic regimes, and their statistical significance are shown in Table 2. GSE dispersions showed *μ* behaviour independent of concentration across all lubrication regimes, with *μ* standing at 1.2 in the boundary regime, similar to that of buffer (Fig. 1b_1_).

**Table 2:** Friction coefficient (*μ*) of grape seed extract (GSE) lupin protein (LP) and conjugates in different lubrication regimes. Lubrication behaviour of GSE in different concentrations (a), lubrication behaviour of LP in different concentrations (b), influence of the LP concentration on the lubrication behaviour of conjugates (c), and influence of the degree of conjugation (DC) on the lubrication behaviour of conjugates (d).

(a)						
Sample	Boundary lubrication regime		Mixed lubrication regime		Hydrodynamic lubrication regime	
	Mean	SD	Mean	SD	Mean	SD
GSE[0.12]	1.165^a^	0.053	0.552^a^	0.114	0.002^a^	<0.001
GSE[0.5]	1.229^a^	0.042	0.424^a^	0.032	0.003^a^	<0.001
GSE[1]	1.212^a^	0.098	0.333^a^	0.064	0.003^a^	<0.001

(b)						
Sample	Boundary lubrication regime		Mixed lubrication regime		Hydrodynamic lubrication regime	
	Mean	SD	Mean	SD	Mean	SD
LP[1]	0.720^a^	0.034	0.015^b^	0.011	0.002^a^	<0.001
LP[3]	0.788^a^	0.044	0.065^ab^	0.007	0.003^a^	<0.001
LP[5]	0.735^a^	0.055	0.087^ab^	0.053	0.003^a^	<0.001
LP[11]	0.737^a^	0.058	0.151^a^	0.057	0.003^a^	<0.001

(c)						
Sample	Boundary lubrication regime		Mixed lubrication regime		Hydrodynamic lubrication regime	
	Mean	SD	Mean	SD	Mean	SD
LP[1]-GSE	0.772^a^	0.020	0.174^ab^	0.047	0.003^a^	<0.001
LP[3]-GSE	0.767^a^	0.040	0.093^c^	0.022	0.003^a^	<0.001
LP[5]-GSE	0.769^a^	0.051	0.113^bc^	0.011	0.003^a^	<0.001
LP[11]-GSE	0.722^a^	0.110	0.192^a^	0.054	0.003^a^	<0.001

(d)						
Sample	Boundary lubrication regime		Mixed lubrication regime		Hydrodynamic lubrication regime	
	Mean	SD	Mean	SD	Mean	SD
LP-GSE_DC[2]_	0.767^b^	0.040	0.093^c^	0.022	0.003^a^	<0.001
LP-GSE_DC[6]_	0.818^b^	0.042	0.171^b^	0.010	0.003^a^	<0.001
LP-GSE_DC[7]_	0.978^a^	0.086	0.236^a^	0.034	0.003^a^	<0.001
LP-GSE_DC[12]_	0.750^b^	0.059	0.114^c^	0.012	0.003^a^	<0.001

For (c) and (d) GSE concentration was kept constant at 0.12 wt% and for (d) LP concentration was kept constant at 3 wt%. Different letters in each column represent statistical differences with *p < 0.05*.

This suggests that GSE cannot form effective boundary tribofilms in the absence of saliva. The onset of the mixed lubrication regime occurred at lower *η*_*∞*_ *x u* values compared to buffer, indicating a delayed de-wetting effect of GSE or a tribofilm that could partially separate the surfaces. Such difference in the boundary and mixed lubrication behaviour implies a GSE-derived tribofilm that is not lubricious, though it could help in separating contacting surfaces to a marginal extent (*i.e.,* a physical barrier against direct contact of PDMS surfaces) in the mixed regime (Sarkar et al., 2021; Soltanahmadi et al., 2022). The lubrication performance of TA was analyzed as a control to verify whether the lubrication of GSE resembles the behaviour of a purer phenolic compound that is known to be astringent. As shown in Fig. S1 in the Supplementary Information (SI), GSE and TA showed similar *μ* values across all the lubrication regimes (*p < 0.05*).

LP dispersions showed lower *μ* values, compared to buffer, across the lubrication regimes to extents similar to those previously reported (Table 2b) (Kew et al., 2021). The decrease in the boundary *μ* suggests the formation of a lubricious tribofilm separating the contact surfaces effectively and promoting a lower resistance to the relative motion of the contact surfaces. Noteworthy, no difference was found for *μ* values (∼0.7) at the boundary regime with the increase of LP concentration. On the other hand, marginally higher *μ* values were observed at higher concentrations of LP in the mixed regime, which also appeared to delay the onset of the mixed regime. The full reasoning for this observation is not clear, though it might be associated with the topography of the tribofilms at higher concentrations (*e.g.,* patchier) or starvation of the contact interface (large deformation and narrow gap) caused by the confinement of LP at the contact inlet (Cartwright et al., 2024). The LP dispersions showed significantly lower *μ* values as compared to the GSE dispersions in the boundary and mixed regimes, indicating the promising lubricity of LP tribofilms.

The effect of viscosity was further investigated by plotting *μ* curves as a function of the theoretical minimum film thickness (*h*_min_) (Sarkar et al., 2019a; Sarkar et al., 2021; Soltanahmadi et al., 2023) (Fig. S2). The values of the theoretical minimum film thickness (*h*_min_) at the transition between the boundary and the mixed regime, and between the elastohydrodynamic (EHL) and the hydrodynamic regime are also presented in Table S1. GSE curves (Fig. S2a and Table S1) showed no difference in *μ*-*h*_*min*_ despite the increase in concentration. In contrast, the *μ*-*h*_*min*_ for LP dispersions showed a similar concentration dependency to that of the μ - *η*_*∞*_ *x u* curves (Fig. S2b and Table S1). The transition between the boundary and mixed regimes appeared at *h*_min_ values ranging between 9 and 28 nm (comparable to or smaller than the combined surface roughness of PDMS specimens), which were smaller than that of GSE occurring at 30 nm (larger than the combined surface roughness of PDMS specimens). The *h*_min_ at which the transition between the EHL and hydrodynamic regimes appeared were ∼0.9 and ∼0.5 μm for GSE and LP dispersions, respectively (Table S1). Essentially, the tribofilms from LP showed promising lubricity and enhanced the wetting effect compared to GSE tribofilm (Sarkar et al., 2021; Shewan et al., 2020; Soltanahmadi et al., 2023).

#### 3.2.2 Lubrication behaviour of conjugates

GSE at 0.12 wt% was selected to produce conjugates of LP and GSE. This concentration was selected based on the previous study of Sun et al. (2013) involving the investigation of proanthocyanidins interactions with human saliva.

**Fig. 2:**
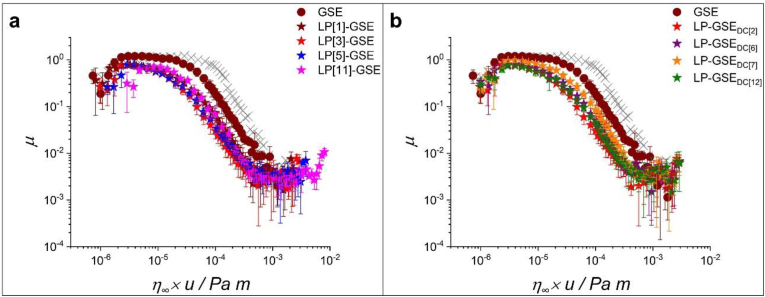
Friction curves normalized to the viscosity at a shear rate of 10^3^ s^−1^ (*η*_∞_) for LP-GSE conjugates showing the influence of (a) LP concentration and (b) degree of conjugation (DC) keeping the concentration of LP constant. GSE concentration was kept constant at 0.12 wt%. The gray symbol is the curve for water. Error bars represent standard deviations (n = 3 × 3).

*Influence of LP:GSE ratio in the lubrication performance of conjugates.* As can be seen in Fig. 2a, the LP-GSE conjugate dispersions at LP:GSE ratios of 1:0.12, 1:0.04, 1:0.02, 1:0.01 w/w overlapped. Conjugation of LP to GSE reduced the *μ* in the boundary (up to 34%) and mixed (up to 83%) regimes compared to GSE alone, indicating the ability of LP conjugation to improve the lubrication of GSE (*p < 0.05*, Table 2c).

The lowest LP:GSE ratio tested (LP[1]-GSE) showed marginally higher *μ* than LP[1] in the mixed lubrication regimes (*p < 0.05*, two-sample *t*-test, data not shown), which can be attributed to the exclusion effect of GSE conjugation on protein film formation or inferior lubricity of conjugate tribofilms. This suggests a critical LP:GSE ratio is required to achieve a lubricity degree in the order of the lubricity of protein alone. With the increased LP:GSE ratio, LP[3]-GSE, LP[5]-GSE, and LP[11]-GSE samples showed similar (*p > 0.05*) *μ* values to the respective LP-only (*i.e.*, LP[3], LP[5], and LP[11], respectively) in the boundary and EHL lubrication regimes with a slightly higher (*p < 0.05*) *μ* values in the mixed regime (Table 2c). It can be, therefore, inferred that tribofilms formed by LP-GSE conjugates provide similar lubricity to that of tribofilms from LP alone at the extreme solid-solid contact (*i.e.*, boundary regime), and in the EHL regime dominated by the viscous behaviour of LP. As mentioned above, the exclusion effect of GSE conjugation on protein film formation appears to induce higher *μ* in the mixed regime.

*Influence of reaction time and degree of conjugation (DC) in the lubrication performance of conjugates.* It is important to highlight that studies on the effect of reaction time on covalent conjugation between proteins and polysaccharides have already been performed, showing considerable time dependency (Chen et al., 2019; Dursun Capar and Yalcin, 2021; Gao et al., 2024). However, to the best of our knowledge, the study of the evolution of covalent bond formation between protein and phenolic compounds over time has not been studied so far. Most of the studies available performed the alkaline reaction up to 24 h, considering that the reaction has reached an equilibrium by that time (Liu et al., 2016; Yan et al., 2020; Zhao et al., 2021), but studies confirming that longer periods would not result in higher values of DC are not available to date. Also, the influence of DC in protein-phenolic conjugates on tribological performance remains unexplored. LP[3]-GSE was chosen to study the influence of the reaction time on the lubrication performance of the conjugates. LP at 3 wt% was selected as it was the concentration just above the critical ratio required to achieve a lubricity degree matching that of LP protein alone (Tables 2b and 2c).

Reaction times of 4, 24, 48, and 168 h resulted in samples with DC values of 2, 6, 7, and 12%, respectively, accompanied by a change in color (Fig. S3). The increase in DC resulted in no statistically significant difference in *μ* in the boundary, mixed, and hydrodynamic regimes (*p > 0.05*, Fig. 2b–Table 2d) except for LP-GSE_DC[7],_ which showed higher *μ* compared to other dispersions in Fig. 2b. Overall, a significant difference in the lubrication performance of LP-GSE, as a result of DC, can be ruled out within the DC obtained in this study.

#### 3.2.3 Interaction of sole components and LP-GSE conjugate with human saliva

**Fig. 3:**
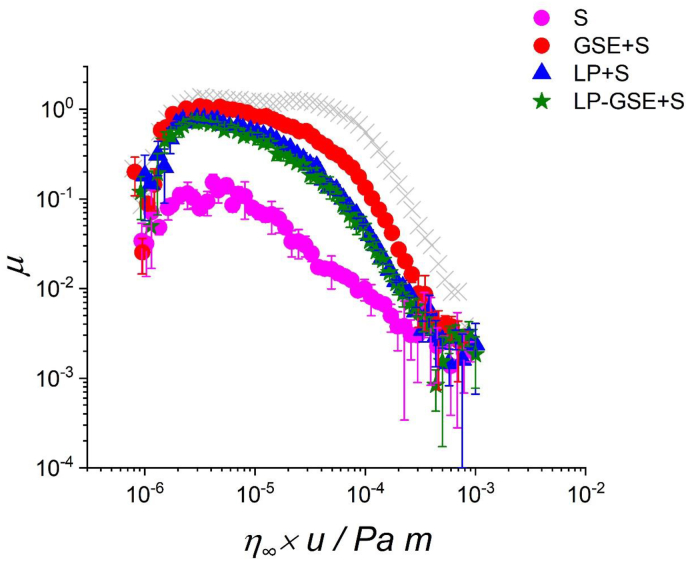
Friction curves normalized to the viscosity at a shear rate of 10^3^ s^−1^ (*η*_∞_) for saliva (S), and saliva mixed with grape seed extract (GSE + S) lupin protein (LP + S) and lupin protein-grape seed extract conjugate (LP-GSE + S). GSE concentration was kept constant at 0.12 wt% and LP concentration was kept constant at 3 wt%. The gray symbol is the curve for buffer. Error bars represent standard deviations (n = 3 × 3).

Fig. 3 shows the lubrication performance of the mixtures of human saliva with LP (LP + S), GSE (GSE + S), and LP-GSE (LP-GSE + S). The solution of human saliva (S) showed *μ* values close to 0.1 in the boundary regime, an order of magnitude lower compared to water and similar to those previously reported (Table 3) (Sarkar et al., 2019a). GSE + S promoted a visible precipitate (Brandão et al., 2014; Sun et al., 2013)), which reflected in a loss of lubricity of S with a 20-fold and 9-fold increase in *μ* in the mixed (5 × 10^−5^ Pa m) and boundary regimes, respectively (Table 3).

**Table 3:** Friction coefficient (*μ*) of grape seed extract (GSE) lupin protein (LP) and lupin protein-grape seed extract conjugate (LP-GSE) mixed with human saliva (S) in different lubrication regimes.

Sample	Boundary lubrication regime		Mixed lubrication regime	
	Mean	SD	Mean	SD
Saliva	0.117^c^	0.025	0.016^c^	0.007
GSE + Saliva	1.053^a^	0.038	0.330^a^	0.028
LP + Saliva	0.766^b^	0.054	0.138^b^	0.018
LP-GSE + Saliva	0.723^b^	0.066	0.138^b^	0.038

GSE concentration was kept constant at 0.12 wt% and LP concentration was kept constant at 3 wt%. Different letters in each column represent statistical differences with *p < 0.05*.

On the other hand, adding LP to saliva did not show the formation of such visible aggregates. The *μ* values in the mixed and boundary regimes for LP + S were similar to those of LP (and higher than those of S), suggesting that LP dominated the lubrication behaviour in LP + S mixtures. This can be attributed to a competition in surface adsorption between salivary proteins and LP (discussed in the next section).

Similar to LP + S, LP-GSE + S showed no visible precipitation with the *μ* curves for LP + S and LP-GSE + S overlapping. These observations confirm the role of conjugation in hindering undesirable interactions between GSE and S. Addition of S did not appear to alter the dominant influence of LP in driving the lubrication performance. Further, the *μ* values observed for LP + S and LP-GSE + S in the boundary (*μ* ∼0.7) and mixed (*μ* ∼0.1) regimes were significantly lower (p < *0.05*) than those for GSE (*μ* in boundary ∼1.0, and *μ* in mixed ∼0.3) - thanks to the effective lubricity of LP or LP-GSE tribofilms. The promising effect of conjugation requires validation through human sensory trials in future works.

### 3.3 Surface adsorption measurements by QCM-D

QCM-D offers information on the surface adsorption of biopolymeric dispersions, which can be exploited to explain tribological observations (Akgonullu et al., 2023; Nimaming et al., 2023; Soltanahmadi et al., 2022).

#### 3.3.1 Adsorption of GSE, LP, and LP-GSE in the absence of saliva

**Fig. 4:**
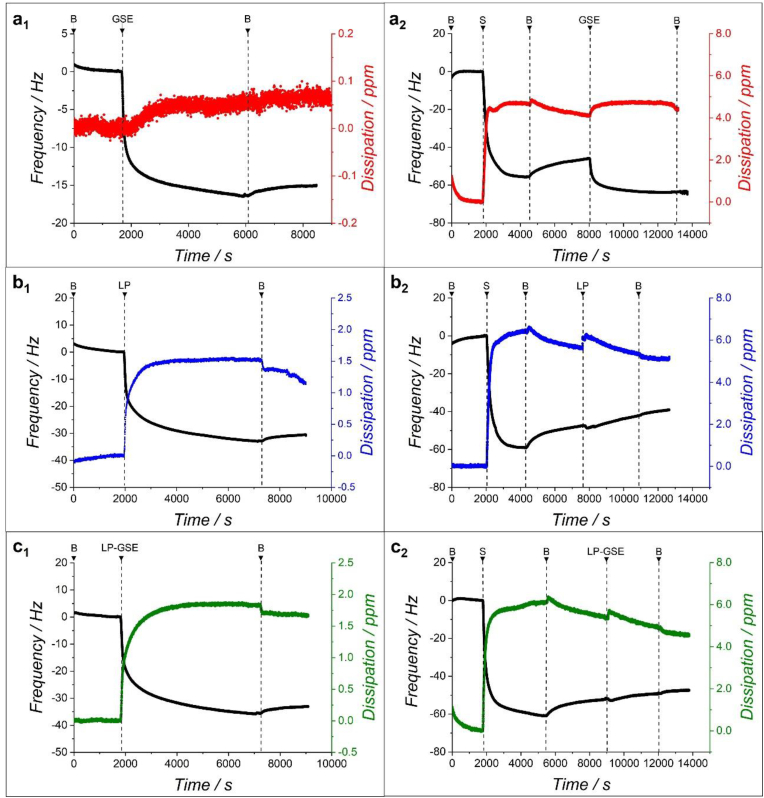
Mean frequency (5th overtone shown) of grape seed extract (GSE) (a_1_), lupin protein (LP) (b_1_), and lupin protein-grape seed extract conjugate (LP-GSE) (c_1_) and saliva (S) interacting with GSE (a_1_), LP (b_2_) and LP-GSE (c_2_) on PDMS-coated sensors, with B implying the HEPES buffer.

The shift in dissipation (*ΔD*) and frequency (*Δf*) as a function of measurement time obtained for GSE, LP, and LP-GSE conjugate are shown in Fig. 4. Since the sample LP[3]-GSE_DC[2]_ showed the lowest *μ* values (Table 2d), that formulation was selected as the LP-GSE conjugated system in QCM-D experiments. Buffer was used as a baseline, followed by the addition of the dispersions, which in all cases promoted a substantial reduction in frequency, indicating surface adsorption. In all samples, the decrease in *f* was accompanied by an increase in dissipation, showing the building up of viscoelastic layers. The last step of rinsing with the buffer promoted a slight increase in *f* in all samples (Fig. 4), indicating the release of a small amount of loosely adsorbed mass occurred irrespective of the samples tested.

**Fig. 5:**
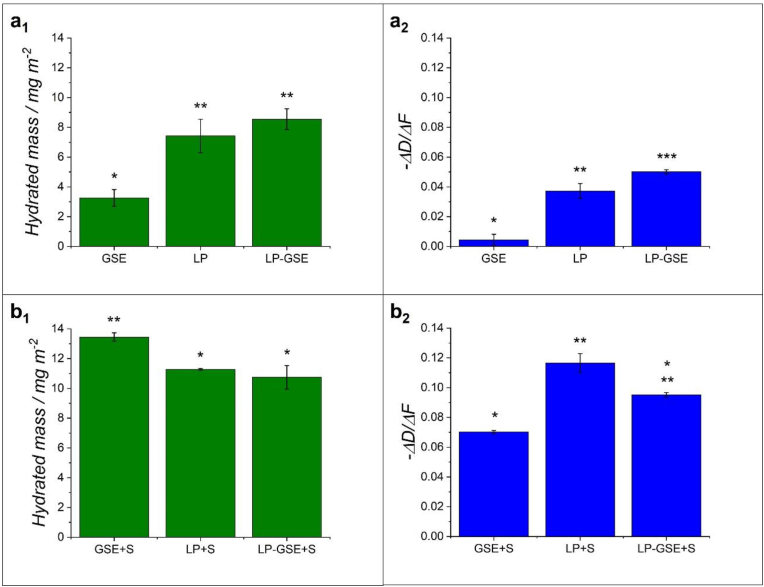
Hydrated mass of (a_1_) grape seed extract (GSE), lupin protein (LP) and lupin protein-grape seed extract conjugate (LP-GSE) films and (a_2_) saliva (S) composite films with GSE (GSE + S), LP (LP + S) and LP-GSE (LP-GSE + S) on PDMS-coated hydrophobic sensors using QCM-D. Hydrated film viscoelasticity (*-ΔD/Δf*) of (a_2_) GSE, LP, and LP-GSE and (b_2_) GSE + S, LP + S, and LP-GSE + S. Error bars indicate standard deviation for triplicate experiments (n = 3). The asterisk represents significant difference (*p < 0.05*).

In particular, GSE (Fig. 4a_1_) showed a significantly smaller *Δf* compared to LP (Fig. 4b_1_) and LP-GSE (Fig. 4c_1_), indicating limited mass adsorption (Fig. 5a_1_). The inferior mass adsorption of GSE can be attributed to its smaller molecular weight (proanthocyanidins) compared to lupin protein fractions (Czochanska et al., 1980; Rumiyati et al., 2012) and/or its limited affinity to hydrophobic PDMS surfaces. Interestingly, GSE showed slightly higher mass adsorption than TA (Fig. S4 and Fig. S5) (*p < 0.05*), unlike the identical tribological behaviour (Fig. S1, *p > 0.05*). Of more importance, GSE showed a smaller *-ΔD/Δf* as compared to LP and LP-GSE (Fig. 5a_2_), indicating a relatively more rigid nature of GSE adsorbed-film (indicates more rigid films (Xu et al., 2020)), which could be due to differences in particle size and particle-surface interactions.

LP showed *f* and *D* profiles (Fig. 4b_1_) similar to previous studies (Kew et al., 2021; Liamas et al., 2023). LP-GSE (Fig. 4c_1_) showed a similar hydrated mass (Fig. 5a_1_) but a higher *-ΔD/Δf* (Fig. 5a_2_) as compared to LP. The more viscous nature of the conjugate film can be attributed to a reduction in protein-protein interactions induced by GSE, leading to a less cohesive film (Francisco et al., 2023). Overall, the improved adsorption of behaviour and film formation of LP and LP-GSE compared to GSE corroborate the better lubrication performance of LP and LP-GSE observed in the tribology section (Fig. 1, Fig. 2).

#### 3.3.2 Interactions between GSE, LP, and LP-GSE with human saliva

Fig. 4 shows the sequence of human saliva (S) deposition onto the PDMS-coated sensors, followed by the adsorption of GSE, LP, or LP-GSE. A fast change in *Δf* exhibited the adsorption of S onto PDMS-coated sensors, containing a significant amount of loosely attached molecules removed following rising with buffer, leaving a viscous film (high *ΔD)*. The following injection of GSE (Fig. 4a_2_) caused a significant decrease in *Δf,* suggesting a clear interaction between GSE molecules and the adsorbed saliva film. After rinsing with buffer, no alteration in *Δf* was recorded, indicating a nonreversible strong bonding between GSE and proteins from saliva, which led to a hydrated adsorbed mass of about 13 mg m^−2^ (Fig. 5b_1_).

In contrast, the injection of LP into the previously adsorbed saliva film (Fig. 4b_2_) promoted first a marginal decrease in *Δf* followed by a constant increase in *Δf* even throughout the buffer rising, not reaching a plateau during the study window. This result suggests that instead of interacting with proteins in saliva, LP may have competed with salivary film over surface adsorption to hydrophobic PDMS surface, depleting previously adsorbed saliva proteins from the surface. This behaviour may explain the governing influence of LP on the lubrication performance over human saliva observed in the tribology study (Fig. 3).

Similarly, LP-GSE showed a small degree of interaction with the saliva protein film (slight *Δf* decrease followed by a continuous increase) (Fig. 4c_2_) but, differently to LP, a plateau was reached after rising with buffer, suggesting a less pronounced change into the film composition over time, or competition over surface adsorption. Due to the lower interaction of LP and LP-GSE with the proteins from saliva, a lower degree of surface-adsorbed mass was recorded compared to GSE (Fig. 5b_1_). These low interactions also explain the higher values of *-ΔD/Δf* verified for LP and LP-GSE in comparison to GSE (Fig. 5b_2_), suggesting the formation of more viscous and looser films. These results confirm the efficacy of conjugating LP to GSE on hindering its interaction with human saliva, leading to limited lubrication loss.

## 4 Conclusions

The lubrication performance and surface adsorption of grape seed extract, lupin protein, and their covalent conjugates in the absence and presence of human saliva (*ex vivo*) were investigated to shed light on the effects of formulation and covalent conjugation on astringency perception of phenolic compounds. Soft tribology and QCM-D results showed that in the absence of saliva, grape seed extract performed as a poor lubricator in the boundary lubrication regime due to the formation of a fragile film that was easily depleted from the contact under shear. In contrast, lupin protein reduced friction in all lubrication regimes, confirming its ability to reduce friction even at low concentrations. Both tribological studies on the variation of lupin protein:grape seed extract ratio and degree of conjugation demonstrated that the covalent conjugation between lupin protein and grape seed extract hinders the ability of lupin moieties to reduce friction in the boundary regime. Still, by tuning lupin protein:grape seed extract ratio and degree of conjugation towards limiting conjugation to retain some degree of free lupin protein molecules in the continuum, the conjugates can present similar lubrication performance to that of pure lupin protein dispersions. In the presence of saliva, GSE showed great interaction with salivary proteins, leading to precipitation and loss of lubrication, which was confirmed by the non-reversible bonding between GSE molecules and the saliva film verified by QCM-D measurements. The covalent conjugation of LP to GSE proved to be effective in attenuating GSE's interaction with human saliva, preventing salivary proteins from precipitating. However, LP seemed to compete with proteins from human saliva on the lubrication of surfaces, leading to inferior lubrication of LP and LP-GSE saliva mixtures. Our results highlight the application of covalent conjugation of phenolics with plant proteins as a useful strategy to address astringency, which needs validation *via* sensory trials.

## CRediT authorship contribution statement

**Cristhian Rafael Lopes Francisco:** Conceptualization, Methodology, Investigation, Data curation, Formal analysis, Writing – review & editing, Visualization, Writing – original draft, Writing – review & editing, Funding acquisition. **Siavash Soltanahmadi:** Conceptualization, Data curation, Methodology, Writing – review & editing, Supervision. **Tatiana Porto Santos:** Conceptualization, Writing – review & editing. **Rosiane Lopes Cunha:** Conceptualization, Writing – review & editing, Supervision, Funding acquisition. **Anwesha Sarkar:** Conceptualization, Project administration, Writing – review & editing, Supervision, Funding acquisition.

## Declaration of competing interest

No conflicts of interests.

## Data Availability

Data will be made available on request.

## References

[bib1] Akgonullu D.Z., Murray B.S., Connell S.D., Fang Y., Linter B., Sarkar A. (2023). Synthetic and biopolymeric microgels: review of similarities and difference in behaviour in bulk phases and at interfaces. Adv. Colloid Interface Sci..

[bib2] Bongaerts J.H.H., Rossetti D., Stokes J.R. (2007). The lubricating properties of human whole saliva. Tribol. Lett..

[bib3] Brandão E., Soares S., Mateus N., De Freitas V. (2014). In vivo interactions between procyanidins and human saliva proteins: effect of repeated exposures to procyanidins solution. J. Agric. Food Chem..

[bib4] Camboim Rockett F., de Oliveira Schmidt H., Schmidt L., Rodrigues E., Tischer B., Ruffo de Oliveira V., Lima da Silva V., Rossini Augusti P., Hickmann Flôres S., Rios A. (2020). Phenolic compounds and antioxidant activity in vitro and in vivo of Butia and Opuntia fruits. Food Res. Int..

[bib5] Cartwright B., Xu Y., Stokes J.R. (2024). Substrate and fluid film mechanics in rolling-sliding soft contact tribology. Tribol. Int..

[bib6] Chantal M., Sarni-Manchado P., Lefebvre S., Cheynier V., Moutounet M. (2003). Influence of fining with plant proteins on proanthocyanidin composition of red wines. Am. J. Enol. Vitic..

[bib7] Chen W., Lv R., Wang W., Ma X., Muhammad A.I., Guo M., Ye X., Liu D. (2019). Time effect on structural and functional properties of whey protein isolate-gum acacia conjugates prepared via Maillard reaction. J. Sci. Food Agric..

[bib8] Chlif N., Bouymajane A., Oulad El Majdoub Y., Diouri M., Rhazi Filali F., Bentayeb A., Altemimi A.B., Mondello L., Cacciola F. (2022). Phenolic compounds, in vivo anti-inflammatory, analgesic and antipyretic activities of the aqueous extracts from fresh and dry aerial parts of Brocchia cinerea (Vis.). J. Pharmaceut. Biomed. Anal..

[bib9] Church F.C., Swaisgood H.E., Porter D.H., Catignani G.L. (1983). Spectrophotometric assay using o-phthaldialdehyde for determination of proteolysis in milk and isolated milk proteins. J. Dairy Sci..

[bib10] Cosme F., Ricardo-Da-Silva J.M., Laureano O. (2007). Protein fining agents: characterization and red wine fining assays. Ital. J. Food Sci..

[bib11] Czochanska Z., Foo L.Y., Newman R.H., Porter L.J. (1980). Polymeric proanthocyan id ins. Stereochemistry, structural units, and molecular weight. J. Chem. Soc., Perkin Trans..

[bib63] de Vicente J., Stokes J., Spikes H. (2005). The frictional properties of newtonian fluids in rolling–sliding soft-EHL contact. Tribol Lett.

[bib12] Devezeaux de Lavergne M., van Delft M., van de Velde F., van Boekel M.A.J.S., Stieger M. (2015). Dynamic texture perception and oral processing of semi-solid food gels: Part 1: comparison between QDA, progressive profiling and TDS. Food Hydrocolloids.

[bib13] Duan J., Li Y., Gao H., Yang D., He X., Fang Y., Zhou G. (2020). Phenolic compound ellagic acid inhibits mitochondrial respiration and tumor growth in lung cancer. Food Funct..

[bib14] Duranti M., Consonni A., Magni C., Sessa F., Scarafoni A. (2008). The major proteins of lupin seed: characterisation and molecular properties for use as functional and nutraceutical ingredients. Trends Food Sci. Technol..

[bib15] Dursun Capar T., Yalcin H. (2021). Protein/polysaccharide conjugation via Maillard reactions in an aqueous media: impact of protein type, reaction time and temperature. Lebensm. Wiss. Technol..

[bib16] Francisco C.R.L., Santos T.P., Cunha R.L. (2023). Nano and micro lupin protein-grape seed extract conjugates stabilizing oil-in-water emulsions. Food Hydrocolloids.

[bib17] Gao K., Xu Y., Rao J., Chen B. (2024). Maillard reaction between high-intensity ultrasound pre-treated pea protein isolate and glucose: impact of reaction time and pH on the conjugation process and the properties of conjugates. Food Chem..

[bib18] García-Lafuente A., Moro C., Manchón N., Gonzalo-Ruiz A., Villares A., Guillamón E., Rostagno M., Mateo-Vivaracho L. (2014). In vitro anti-inflammatory activity of phenolic rich extracts from white and red common beans. Food Chem..

[bib19] Gupta M., Dey S., Marbaniang D., Pal P., Ray S., Mazumder B. (2020). Grape seed extract: having a potential health benefits. J. Food Sci. Technol..

[bib20] Hagerman A.E., Riedl K.M., Jones G.A., Sovik K.N., Ritchard N.T., Hartzfeld P.W., Riechel T.L. (1998). High molecular weight plant polyphenolics (tannins) as biological antioxidants. J. Agric. Food Chem..

[bib21] Huang R., Xu C. (2021). An overview of the perception and mitigation of astringency associated with phenolic compounds. Compr. Rev. Food Sci. Food Saf..

[bib22] Kew B., Holmes M., Stieger M., Sarkar A. (2021). Oral tribology, adsorption and rheology of alternative food proteins. Food Hydrocolloids.

[bib23] Küpeli E., Şahin F.P., Çaliş I., Yeşilada E., Ezer N. (2007). Phenolic compounds of Sideritis ozturkii and their in vivo anti-inflammatory and antinociceptive activities. J. Ethnopharmacol..

[bib24] Laguna L., Sarkar A. (2017). Oral tribology: update on the relevance to study astringency in wines. Tribol. Mater. Surface Interfac..

[bib25] Lau D.W., King A.J. (2003). Pre- and post-mortem use of grape seed extract in dark poultry meat to inhibit development of thiobarbituric acid reactive substances. J. Agric. Food Chem..

[bib26] Liamas E., Connell S.D., Sarkar A. (2023). Frictional behaviour of plant proteins in soft contacts: unveiling nanoscale mechanisms. Nanoscale Adv..

[bib27] Liu F., Wang D., Sun C., McClements D.J., Gao Y. (2016). Utilization of interfacial engineering to improve physicochemical stability of β-carotene emulsions: multilayer coatings formed using protein and protein-polyphenol conjugates. Food Chem..

[bib28] Ma W., Guo A., Zhang Y., Wang H., Liu Y., Li H. (2014). A review on astringency and bitterness perception of tannins in wine. Trends Food Sci. Technol..

[bib29] Marangon M., Vincenzi S., Curioni A. (2019). Wine fining with plant proteins. Molecules.

[bib30] Monteleone E., Condelli N., Dinnella C., Bertuccioli M. (2004). Prediction of perceived astringency induced by phenolic compounds. Food Qual. Prefer..

[bib31] Mumtaz M.Z., Kausar F., Hassan M., Javaid S., Malik A. (2021). Anticancer activities of phenolic compounds from Moringa oleifera leaves: in vitro and in silico mechanistic study. Beni-Suef Univ. J. Basic Appl. Sci..

[bib32] Nakamura Y., Tsuji S., Tonogai Y. (2003). Analysis of proanthocyanidins in grape seed extracts, health foods and grape seed oils. J. Health Sci..

[bib33] National Center for Biotechnology Information (2023).

[bib34] Nimaming N., Sadeghpour A., Murray B.S., Sarkar A. (2023). Hybrid particles for stabilization of food-grade Pickering emulsions: fabrication principles and interfacial properties. Trends Food Sci. Technol..

[bib35] Pereira A.P., Ferreira I.C.F.R., Marcelino F., Valentão P., Andrade P.B., Seabra R., Estevinho L., Bento A., Pereira J.A. (2007). Phenolic compounds and antimicrobial activity of olive (olea europaea L. Cv. Cobrançosa) leaves. Molecules.

[bib36] Pradal C., Stokes J.R. (2016). Oral tribology: bridging the gap between physical measurements and sensory experience. Curr. Opin. Food Sci..

[bib37] Quan T.H., Benjakul S., Sae-leaw T., Balange A.K., Maqsood S. (2019). Protein–polyphenol conjugates: antioxidant property, functionalities and their applications. Trends Food Sci. Technol..

[bib38] Ricardo‐da‐Silva J.M., Cheynier V., Souquet J.‐M., Moutounet M., Cabanis J.‐C., Bourzeix M. (1991). Interaction of grape seed procyanidins with various proteins in relation to wine fining. J. Sci. Food Agric..

[bib39] Ricci A., Parpinello G.P., Palma A.S., Teslić N., Brilli C., Pizzi A., Versari A. (2017). Analytical profiling of food-grade extracts from grape (Vitis vinifera sp.) seeds and skins, green tea (Camellia sinensis) leaves and Limousin oak (Quercus robur) heartwood using MALDI-TOF-MS, ICP-MS and spectrophotometric methods. J. Food Compos. Anal..

[bib40] Rudge R.E.D., Fuhrmann P.L., Scheermeijer R., van der Zanden E.M., Dijksman J.A., Scholten E. (2021). A tribological approach to astringency perception and astringency prevention. Food Hydrocolloids.

[bib41] Rumiyati R., James A.P., Jayasena V. (2012). Effect of germination on the nutritional and protein profile of Australian sweet lupin (lupinus angustifolius L.). Food Nutr. Sci..

[bib42] Sarkar A., Andablo-Reyes E., Bryant M., Dowson D., Neville A. (2019). Lubrication of soft oral surfaces. Curr. Opin. Colloid Interface Sci..

[bib43] Sarkar A., Krop E.M. (2019). Marrying oral tribology to sensory perception: a systematic review. Curr. Opin. Food Sci..

[bib44] Sarkar A., Soltanahmadi S., Chen J., Stokes J.R. (2021). Oral tribology: providing insight into oral processing of food colloids. Food Hydrocolloids.

[bib45] Sarkar A., Xu F., Lee S. (2019). Human saliva and model saliva at bulk to adsorbed phases – similarities and differences. Adv. Colloid Interface Sci..

[bib46] Shewan H.M., Pradal C., Stokes J.R. (2020). Tribology and its growing use toward the study of food oral processing and sensory perception. J. Texture Stud..

[bib47] Shrestha S., Hag L. van t, Haritos V.S., Dhital S. (2021). Lupin proteins: structure, isolation and application. Trends Food Sci. Technol..

[bib49] Soltanahmadi S., Murray B.S., Sarkar A. (2022). Comparison of oral tribological performance of proteinaceous microgel systems with protein-polysaccharide combinations. Food Hydrocolloids.

[bib50] Soltanahmadi S., Wang M., Gul M.K., Stribițcaia E., Sarkar A. (2023). Tribology and rheology of potato protein and pectin mixtures and Maillard conjugates. Sustain. Food Protein.

[bib51] Su X.Y., Wang Z.Y., Liu J.R. (2009). In vitro and in vivo antioxidant activity of Pinus koraiensis seed extract containing phenolic compounds. Food Chem..

[bib52] Sun B., Sá M. De, Leandro C., Caldeira I., Duarte F.L., Spranger I. (2013). Reactivity of polymeric proanthocyanidins toward salivary proteins and their contribution to young red wine astringency. J. Agric. Food Chem..

[bib53] Vogelsang-O’Dwyer M., Bez J., Petersen I.L., Joehnke M.S., Detzel A., Busch M., Krueger M., Ispiryan L., O'Mahony J.A., Arendt E.K., Zannini E. (2020). Techno-functional, nutritional and environmental performance of protein isolates from blue lupin and white lupin. Foods.

[bib54] Voigt W.U. (1889). The relationship between the two elasticity constants isotropic body. Wiedemann’s Annalen Der Physik Und Chemie.

[bib55] Vuolo M.M., Lima V.S., Maróstica Junior M.R. (2019). Bioactive Compounds: Health Benefits and Potential Applications.

[bib56] Wang S., Olarte Mantilla S.M., Smith P.A., Stokes J.R., Smyth H.E. (2020). Astringency sub-qualities drying and pucker are driven by tannin and pH – insights from sensory and tribology of a model wine system. Food Hydrocolloids.

[bib57] Wang S., Olarte Mantilla S.M., Smith P.A., Stokes J.R., Smyth H.E. (2021). Tribology and QCM-D approaches provide mechanistic insights into red wine mouthfeel, astringency sub-qualities and the role of saliva. Food Hydrocolloids.

[bib58] Xu D., Deng Y., Han T., Jiang L., Xi P., Wang Q., Jiang Z., Gao L. (2018). In vitro and in vivo effectiveness of phenolic compounds for the control of postharvest gray mold of table grapes. Postharvest Biol. Technol..

[bib59] Xu F., Liamas E., Bryant M., Adedeji A.F., Andablo-Reyes E., Castronovo M., Ettelaie R., Charpentier T.V.J., Sarkar A. (2020). A self-assembled binary protein model explains high-performance salivary lubrication from macro to nanoscale. Adv. Mater. Interfac..

[bib60] Yan Y., Zhu Q., Diao C., Wang J., Wu Z., Wang H. (2020). Enhanced physicochemical stability of lutein-enriched emulsions by polyphenol-protein-polysaccharide conjugates and fat-soluble antioxidant. Food Hydrocolloids.

[bib61] Zembyla M., Liamas E., Andablo-Reyes E., Gu K., Krop E.M., Kew B., Sarkar A. (2021). Surface adsorption and lubrication properties of plant and dairy proteins: a comparative study. Food Hydrocolloids.

[bib62] Zhao T., Huang L., Luo D., Xie Y., Zhang Y., Zhang Y., Jiao W., Su G., Zhao M. (2021). Fabrication and characterization of anchovy protein hydrolysates-polyphenol conjugates with stabilizing effects on fish oil emulsion. Food Chem..

