# Supplementary material for: Addressing astringency of grape seed extract by covalent conjugation with lupin protein
Source: Curr Res Food Sci. 2024 Jun 17;9:100795. doi: 10.1016/j.crfs.2024.100795 (PMC11260025; doi:10.1016/j.crfs.2024.100795)
Supplement: Multimedia component 1 [file mmc1.docx]

**Supplementary Information**

**Addressing astringency of grape seed extract by covalent conjugation with lupin protein**

Cristhian Rafael Lopes Francisco ^1,2^, Siavash Soltanahmadi ^1^, Tatiana Porto Santos ^3^ Rosiane Lopes Cunha ^2^, Anwesha Sarkar ^1,*^

^1^Food Colloids and Bioprocessing Group, School of Food Science and Nutrition, Faculty of Environment, University of Leeds, Leeds, LS2 9JT, UK.

^2^Laboratory of Process Engineering, Department of Food Engineering and Technology, School of Food Engineering, University of Campinas (UNICAMP), Rua Monteiro Lobato 80, 13083-862, São Paulo, Campinas, Brazil.

^3^Laboratory of Food Process Engineering, Wageningen University and Research, Bornse Weilanden 9, 6708, WG, Wageningen, the Netherlands.

^*^Corresponding author. Tel.: +44 (0) 113 343 2748.

E-mail address: A.Sarkar@leeds.ac.uk (A. Sarkar).

**Table S1.** Theoretical minimum film thickness (*h_min_*) at boundary to mixed regime and elastohydrodynamic regime (EHL) to hydrodynamic regime transitions in hydrophobic contacts in presence of various samples.

| Sample | *h_min_* at boundary to mixed regime transition / m | *h_min_* at EHL to hydrodynamic regime transition / m |
| --- | --- | --- |
| GSE[0.12] | 2.93x10^-08^ | 8.62x10^-07^ |
| GSE[0.5] | 2.93x10^-08^ | 8.87x10^-07^ |
| GSE[1] | 3.02x10^-08^ | 9.08x10^-07^ |
| LP[1] | 1.50x10^-08^ | 4.90x10^-07^ |
| LP[3] | 0.86x10^-08^ | 4.68x10^-07^ |
| LP[5] | 1.87x10^-08^ | 4.89x10^-07^ |
| LP[11] | 2.77x10^-08^ | 5.22x10^-07^ |


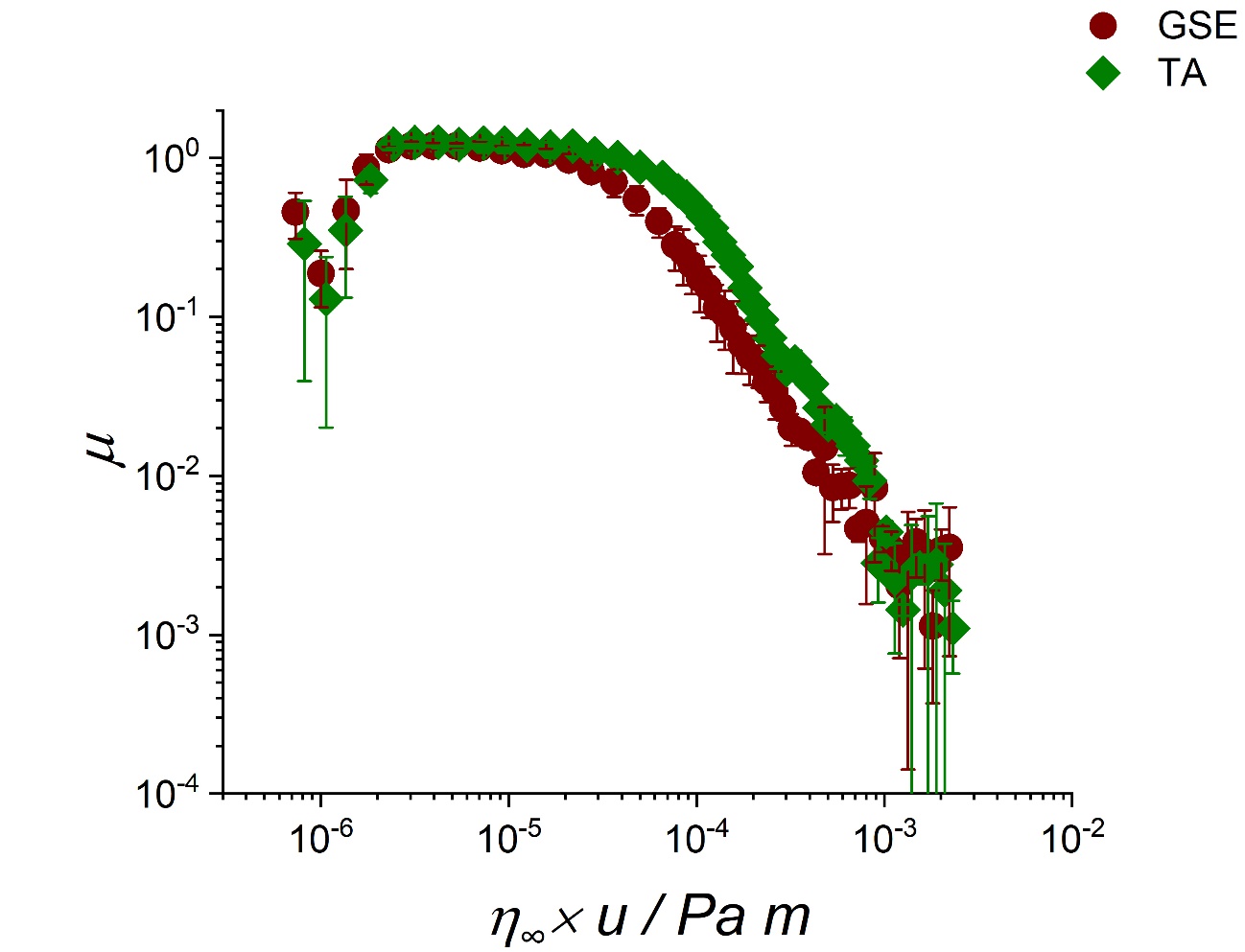


**Fig. S1.** Friction curves normalized to the viscosity at a shear rate of 10^2^ s^−1^ for grape seed extract (GSE) and tannic acid (TA) dispersions at 0.12 wt%. Error bars represent standard deviations (n = 3 × 3).


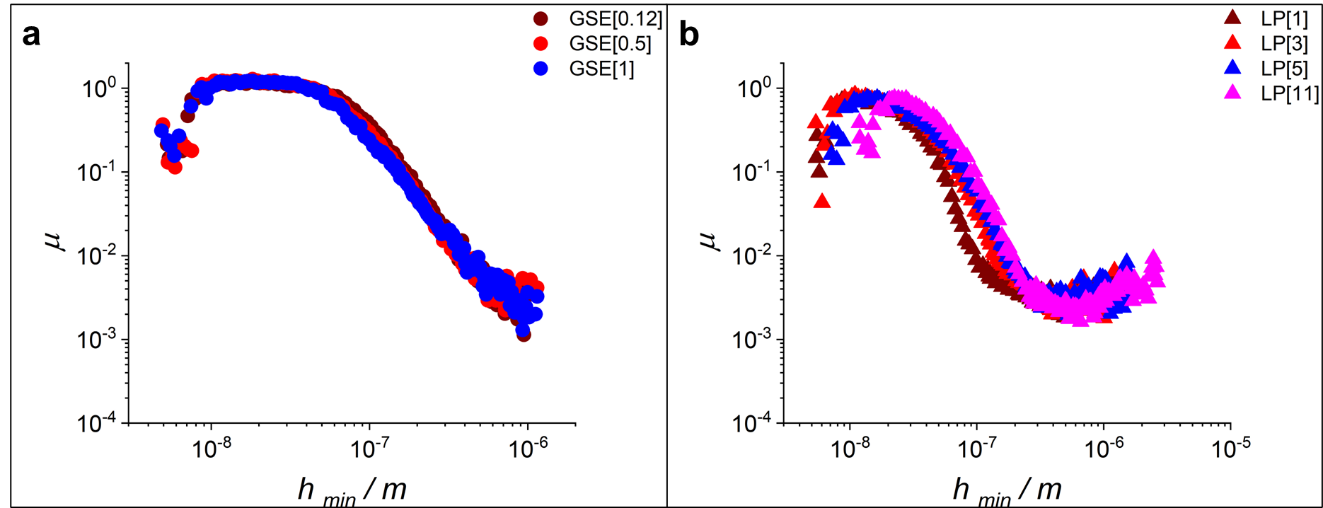


**Fig. S2.** Mean friction coefficient (*μ*) as a function of minimum lubricant film thickness (*h_min_*) for (a) grape seed extract (GSE) dispersions and (b) lupin protein (LP) dispersions*.*


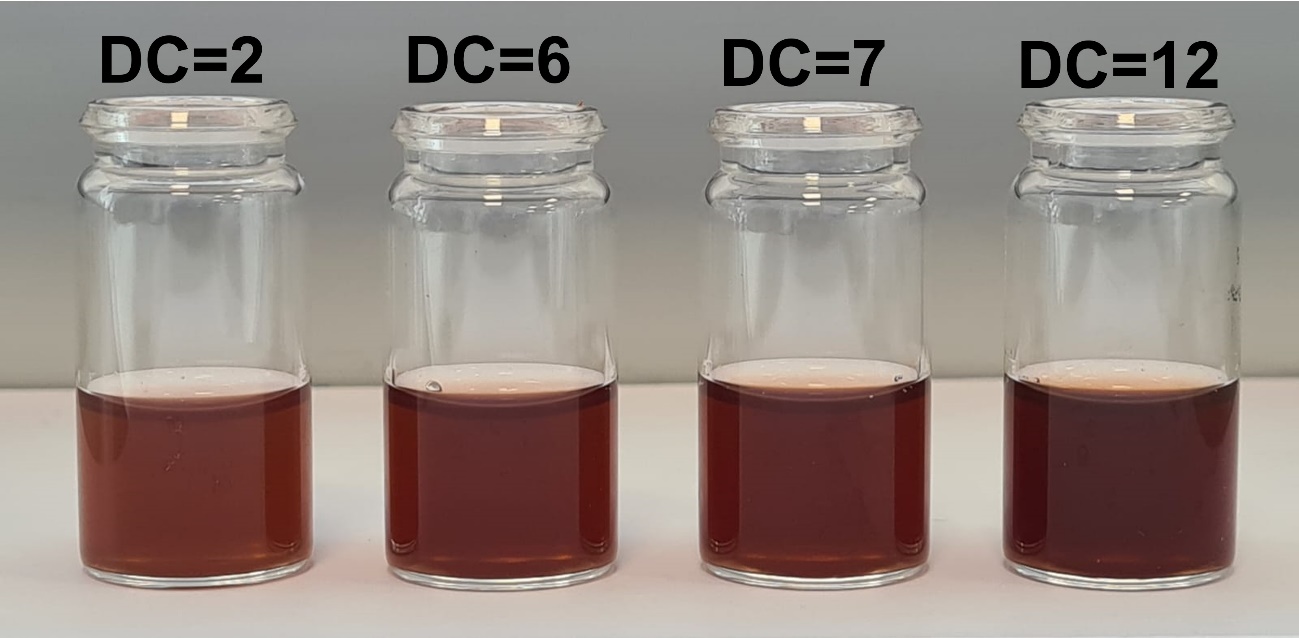


**Fig. S3.** Visual appearance of conjugates LP[3]+GSE produced with different degrees of conjugation (DC).


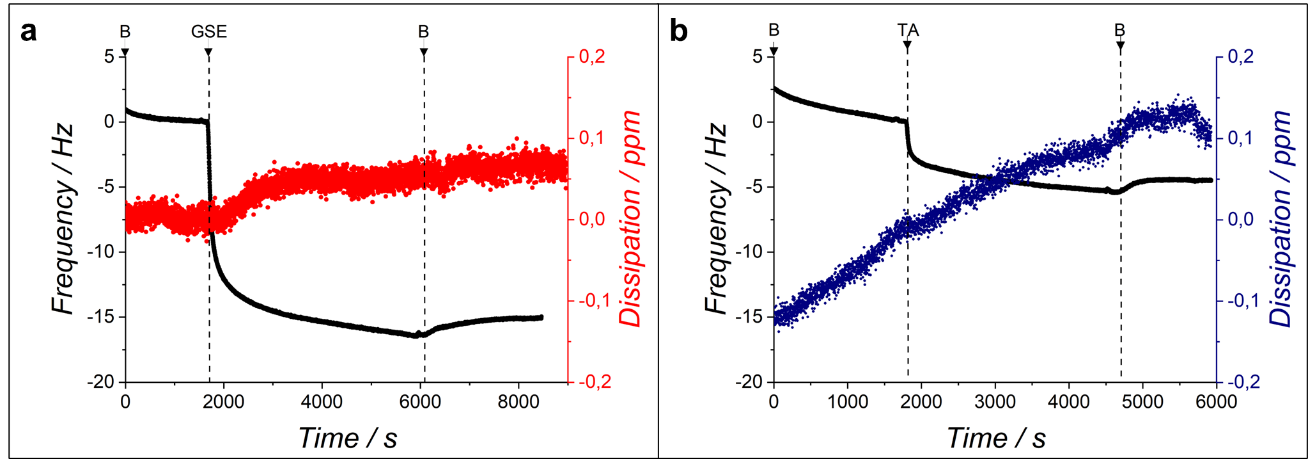


**Fig. S4.** Mean frequency and dissipation (5th overtone shown) of (a) grape seed extract (GSE) and (b) tannic acid (TA) on PDMS-coated hydrophobic sensors, with B implying the HEPES buffer.


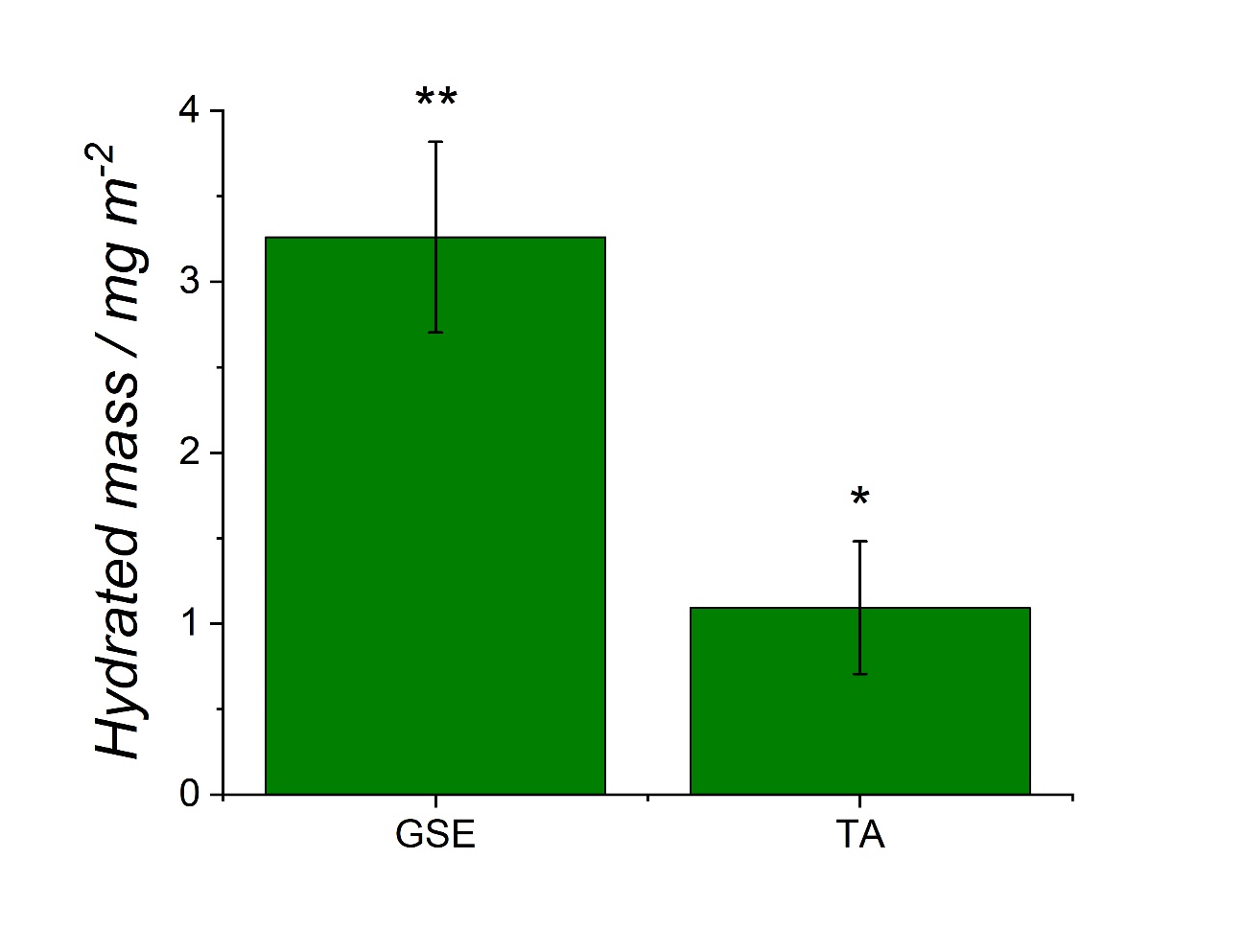


**Fig. S5.** Hydrated mass of GSE and TA on PDMS-coated hydrophobic sensors using QCM-D. Error bars indicate standard deviation for triplicate experiments. The asterisk represents significant difference (p < 0.05).
